# Laboratory Quality Improvement Tutorial Available from CDC

**Published:** 2013-09-06

Application of Laboratory Medicine Best Practices (LMBP) Initiative A-6 Methods for Laboratory Practitioners, a free, online continuing education course, is now available from CDC. This 1-hour tutorial provides a model for the stepwise design and implementation of quality improvement studies, including how these studies can advance evidence-based laboratory medicine. The format presents access to published journal articles, project planning templates, and informative websites. The course is available at https://www.futurelabmedicine.org/tutorials.

